# RNA helicases in RNA decay

**DOI:** 10.1042/BST20170052

**Published:** 2018-01-19

**Authors:** Vanessa Khemici, Patrick Linder

**Affiliations:** Department of Microbiology and Molecular Medicine, Faculty of Medicine, University of Geneva, Geneva, Switzerland

**Keywords:** degradosome, exosome, RNA decay, RNA helicases

## Abstract

RNA molecules have the tendency to fold into complex structures or to associate with complementary RNAs that exoribonucleases have difficulties processing or degrading. Therefore, degradosomes in bacteria and organelles as well as exosomes in eukaryotes have teamed-up with RNA helicases. Whereas bacterial degradosomes are associated with RNA helicases from the DEAD-box family, the exosomes and mitochondrial degradosome use the help of Ski2-like and Suv3 RNA helicases.

## Introduction

All living cells encounter situations where they need to adapt gene expression to changing environmental conditions. The synthesis of new mRNAs to be used for translation and the release of sequestered or translational inactive mRNAs allow the cells to express new proteins. On the other hand, processing and degradation of RNAs not only helps to recycle essential components, but also to shut down expression of genes that are no longer required or would even be detrimental for living under a new condition. Moreover, remnants of processed or aberrant transcripts must rapidly be degraded to avoid the production of useless or even toxic peptides and proteins. Eubacteria, Archaea, and eukaryotes have developed dedicated pathways and complexes to process RNA, check the accuracy of RNAs (surveillance), and feed undesired RNA into exoribonucleases that degrade RNA in a 3′–5′ or 5′–3′ direction. In addition to the ribonucleases, these complexes often contain adaptor proteins such as Hfq (an RNA chaperone), RraA (a regulator of RNase E and DEAD-box helicases), poly(A) polymerase (to render 3′-ends accessible to the 3′–5′ PNPase), or others [1,2]. It is interesting to note that the different RNA degradation machineries all harbour RNA helicases and have been suggested to be invented several times during evolution [3].

RNA helicases are large families of proteins that share up to 12 motifs involved in nucleotide-triphosphate binding, interaction with RNA, and intramolecular contacts [4]. According to differences in their sequence motifs, they can be attributed to various families [5]. In this review, we will describe the interaction of DEAD-box helicases with bacterial degradosomes, RNase R, an RNase carrying an additional helicase activity, the interaction of Ski2-like RNA helicases with eukaryotic exosomes, and the interaction of the Suv3 RNA helicase with the mitochondrial degradosome.

## DEAD-box RNA helicases assist bacterial degradosomes

Helicases are subdivided in several superfamilies, with superfamily 2 containing most of the RNA helicases, including the DEAD-box family. DEAD-box proteins can clearly be distinguished from the other RNA helicases based on the conserved motifs that are involved in ATP binding, RNA binding, and intramolecular interactions [4]. These proteins are ATP-dependent RNA-binding proteins that hydrolyse the ATP once bound to RNA. Upon release of P_i_, the helicase will have reduced affinity for RNA and dissociate again. The binding to RNA induces a bending of the substrate that is incompatible with a double-stranded RNA and therefore induces a local, non-processive, unwinding [6–8]. The freed single-stranded RNA can then anneal with a complementary nucleic acid or be bound (or digested) by a protein. Although the core domain, containing the conserved motifs, may confer some specificity to these highly conserved proteins, as in the case of the short DEAD-box protein eIF4A [9], the specificity of most DEAD-box RNA helicases is given by the N- and/or C-terminal extensions. In addition to the expected local unwinding activity, DEAD-box proteins can also dissociate proteins from RNA or even anneal two complementary molecules and thereby be involved in strand exchange reactions [10,11].

Many DEAD-box proteins were described for bacteria, although the number of different RNA helicases in bacteria is clearly smaller than in eukaryotic cells and, in general, they are not essential under laboratory growth conditions [12]. Indeed, even the deletion of all DEAD-box protein genes from *Escherichia coli* (five genes) or *Bacillus subtilis* (four genes) are viable and do not show drastic synthetic enhancements at 37°C [13,14], indicating that they do not perform redundant functions. However, at low temperature (16°C), the quadruple mutant in *B. subtilis* was unable to grow [13]. So far, bacterial RNA helicases of the DEAD-box protein family were found to be involved in translation initiation, ribosome biogenesis, and RNA decay (for review, see ref. [15]). The involvement of RNA helicases in the degradation of RNAs has been intensively studied over the years and much is known about their requirement and the interaction with other proteins [16].

**Figure 1.**
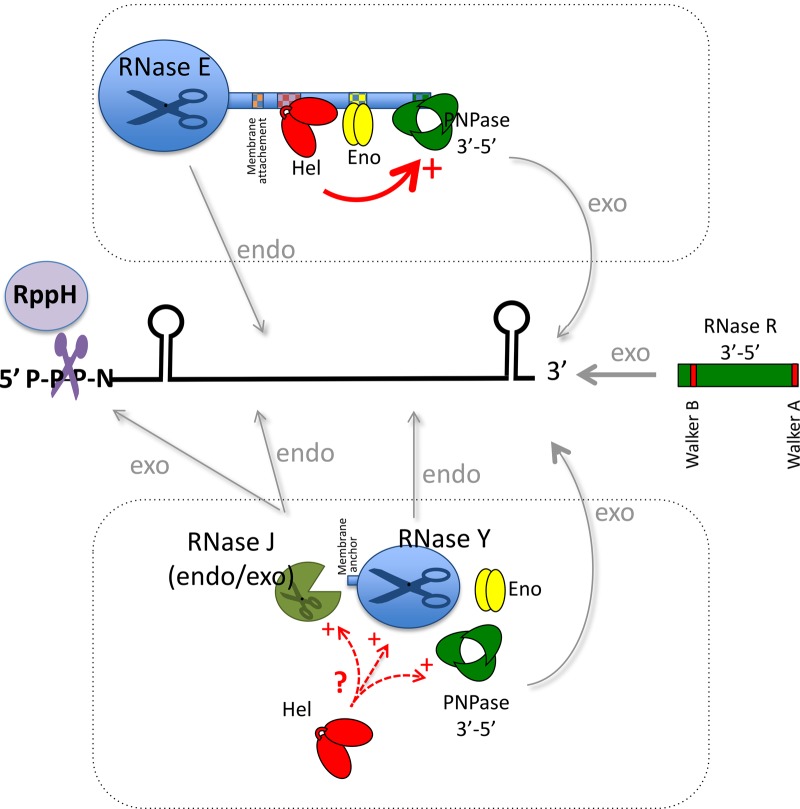
The bacterial degradosomes. RNA degradosomes from different bacteria use similar mechanisms to degrade RNA. In *E. coli,* degradation of most RNAs begins by an endoribonucleotytic cleavage, followed by the fast removal of intermediates by 3′–5′ exoribonucleases, which may be assisted by RNA helicases to remove secondary structures, inhibitory for PNPase. The initiating endoribonucleolytic cleavage step is favoured by RppH removing the 5′ triphosphate to stimulate RNase E activity. The general mRNA degradation pathway is thought to be similar in Gram-positive bacteria, but is achieved by a different set of enzymes as for example the 5′–3′ exoribonuclease RNase J and the endoribonuclease RNase Y. These enzymes, along with the RNA helicase, have been proposed to associate into a degradosome.

The *E. coli* degradosome was the first RNA degradative complex discovered [17]. Its major components are RNase E (the essential endoribonuclease), PNPase (a 3′–5′ exoribonuclease), enolase (a glycolytic enzyme), and RhlB (a DEAD-box RNA helicase) [17,18] (Figure 1). The assembly of the four components relies on strong interactions with the unstructured C-terminal end of RNase E, whereas its N-terminal domain bears its catalytic activity. RNase E is involved in maturation of stable RNAs and mRNAs, but also plays a major role in mRNA decay and sRNA-mediated regulation.

In the initial discovery of the RNA degradosome that identified RNase E and PNPase, the authors have shown that *in vitro* degradation of a structured RNA required the presence of ATP, which was later shown to be used by RhlB [18]. A minimal degradosome composed of RNase E, the PNPase, and the RhlB helicase was shown to be able to degrade structured RNA if the PNPase and the RNA helicase are associated together through the RNase E scaffolding domain [19]. *In vitro* RhlB requires its interaction with RNase E to be stimulated in its ATPase activity [20]. Thus, activity of RhlB is controlled by RNase E and involved in stimulation of the PNPase. Moreover, *in vivo* work showed that RhlB does, indeed, facilitate the degradation of highly structured intermediates by the PNPase, although the situation become less stringent than *in vitro*, since the 3′ polyadenylation of RNAs by poly(A) polymerase PAP allows the PNPase to have several attempts to degrade the structured substrate [21]. In a pioneering work, Bernstein et al. [22] have shown that all components of the degradosome are important for decay, but that the *rhlB* and *eno* mutants significantly affect specific RNAs such as operons involved in the utilisation of diverse carbon sources in an enolase mutant, or the *nuo* operon (NADH dehydrogenase subunits) and the *sdh* operon (succinate dehydrogenase) in an *rhlB* mutant. However, the bulk of RNAs was only slightly affected, with median half-lives increasing from 3.7 to 4.0 min in the *rhlB* and *eno* mutants when compared with wild type.

Although RhlB is considered the main RNA helicase in the *E. coli* degradosome, it has been reported that the RNA helicase CsdA can associate with the degradosome at low temperatures. Moreover, in a *csdA* mutant, a reporter mRNA is stabilised at 22°C, but not at 37°C, indicating that this DEAD-box protein functions in a ‘cold-shock’ degradosome [23]. Similarly, it was shown that in a minimal degradosome, RhlE can functionally replace RhlB [24] (Table 1). In this context, it is interesting to note that some environmental bacteria, such as *Vibrio* or *Shewanella*, have multiple genes that resemble *rhlE* [15].

**Table 1:** Bacterial degradosomes as described in the literature

Organism	Helicase	Endo	5′–3′	3′–5′		
*Escherichia coli*	RhlB	RNase E		PNPase	Enolase	[18]
	CsdA (in cold)	RNase E		PNPase	Enolase	[23]
	RhlE (*in vitro*)	RNase E		PNPase	Enolase	[24]
*Streptomyces coelicolor*		RNase E		PNPase		[73]
*Pseudomonas syringae* Lz4W	RhlE	RNase E		RNase R		[74]
*Vibrio angustum* S14	RhlB	RNase E		PNPase	Enolase	[75]
*Caulobacter crescentus*	RhlB RhlE	RNase E		PNPase, RNase D	Aconitase	[76,77]
*Pseudoalteromonas haloplanktis*	RhlB	RNase E		PNPase		[78]
*Anabaena* PCC7120		RNase E		PNPase		[79]
*Synechocystis* PCC6803	CrhR	RNase E	RNase J	PNPase		[37,79]
*Yersinia pseudotuberculosis*	RhlB	RNase E		PNPase	Enolase	[80]
*Rhodobacter capsulatus*	ORF 1970 ORF 4133	RNase E				[81]
*Helicobacter pylori*	RhpA		RNase J			[32]
*Bacillus subtilis*	CshA	RNase Y	RNase J	PNPase	Enolase	[25]
*Staphylococcus aureus*	CshA	RNase Y	RNase J	PNPasse	Enolase	[26,27]

Whereas the RNase E-dependent degradosome is present in many bacteria, some bacteria do not encode RNase E, but nevertheless have similar RNA-degrading machines. The Firmicutes *B. subtilis* and *Staphylococcus aureus* have a degradosome composed of RNase J (a 5′–3′ exo- and endonuclease), RNase Y (endonuclease), the PNPase (3′–5′ exoribonuclease), the enolase, the phosphofructokinase, and last but not least CshA (an RNA helicase) [25–27]. Although the different Firmicute degradosomes are similar, the reported interactions between components may slightly differ, which could be attributed to species or methodological differences. Moreover, the interaction between the various components of the firmicute degradosome seems not to be as strong as in *E. coli*, and it is not clear yet whether these interactions are essential to co-ordinate their activities. Nevertheless, we could show that in *S. aureus*, some mRNAs are stabilised in the absence of CshA, as, for example, the *agrBDCA* mRNA encoding the quorum sensing system resulting in increased levels of RNA III, a readout of the *agr* system [27,28]. RNAIII is a highly structured small RNA that binds certain mRNAs and thereby occludes ribosome-binding sites (RBS) by complementary to the RBS, or liberates RBS by opening secondary structures. This results in decreased surface protein and increased secreted protein expression, leading to decreased biofilm formation and increased haemolysis [28–30]

## Bacterial degradosomes associate with the membrane and ribosomes

The *E. coli* degradosome was found to be associated with ribosomes and polysomes [31], which may account for either a co-translational mRNA degradation activity, the salvage of stalled ribosome, or required for sRNA-mediated regulation leading to the coupling of translation inhibition and RNA degradation. Interestingly, the *Helicobacter pylori* degradosome, which is only composed of an RNase J and an RNA helicase, was also found associated with polysomes [32] and, in *B. subtilis*, it was shown that CshA interacts with ribosomal proteins [13]. Moreover, RNase J was originally found to be associated with ribosomes [33], although a *bona fide* interaction of the Firmicute degradosome awaits confirmation. Overall, the association with ribosomes may be a general feature to rapidly degrade faulty or sRNA-targeted mRNAs.

Another manner to regulate RNA decay could be a subcellular localisation within a cell. In *E. coli*, microscopy analysis of the degradosome has shown that it is mainly associated with the cytoplasmic membrane of the bacterium. The interaction with the membrane occurs through a membrane-targeting sequence, MTS, an amphipathic α-helix in RNase E [34]. Interestingly, it was shown that deletion of the MTS affects growth [34], and that the formation of degradosome foci on the membrane becomes more diffuse when the cells were treated with rifampicin to block transcription [35]. In a recent transcriptome analysis, it was shown that mRNAs encoding proteins that are cotranslationally targeted to the membrane are less stable than others as a consequence of the localisation of the degradosme at the membrane [36]. This membrane association is also found in other bacteria, such as in cyanobacteria, where CrhR helicase is localised to the cytoplasmic and thylakoid membranes [37], and in Firmicutes, where RNase Y has a membrane-spanning domain. Thus, this membrane anchoring is presumably part of a regulatory mechanism to avoid uncontrolled RNA degradation as suggested in *S. aureus*, where the deletion of this domain is able to compensate partially for a reduced degradosome activity in the absence of CshA [38].

## All in one: RNase R

RNase R is, like the PNPase and RNase II, a 3′–5′ exoribonuclease, but whereas the two latter are inhibited by secondary structures or dsRNA, RNase R possesses a helicase activity that allows the degradation of double-stranded RNA that presents a 3′ single-stranded extension [39]. In accordance with this proposition, the gene encoding RNase R was isolated as a multicopy suppressor of a cold-sensitive *csdA* mutant [40]. Interestingly, mutations that abolish RNase activity, but not the helicase activity, are still able to complement the absence of CsdA at low temperatures [41]. Biochemical analyses have shown that RNase R possesses an NTP-dependent RNA helicase activity, and sequence analysis revealed Walker A (GKT) and B (VVPDD) motifs for NTP hydrolysis, but otherwise this protein does not share the other motifs typically found in RNA helicases of the DEAD-box family [42]. Moreover, the two motifs are present at unorthodox locations: the Walker A motif is located in the C-terminal region (P-loop 730–737), whereas the Walker B motif is located in the N-terminal region (164–169) of the protein. Nevertheless, mutational analysis showed that these motifs are important for helicase activity, and the motifs are conserved in 88% mesophilic bacteria, but not in thermophilic bacteria. The helicase activity does not require the hydrolytic exoribonuclease activity; however, the nuclease activity on double-stranded RNA requires the helicase activity [43]. Importantly, the helicase activity is required *in vivo* especially at low temperature [44].

It is therefore not surprising that in *E. coli*, a *rnr*(*vacB*), *pnp* double mutant is not viable, since RNase R with its 3′–5′ hydrolytic exonuclease and helicase activities can fulfil a similar role, as the phosphorylase PNPase coupled with the RNA helicase RhlB in the degradosome [45].

## Ski2-like RNA helicases are cofactors of the eukaryotic exosomes

The Ski2-like RNA helicases represent a distinct family of proteins highly conserved in eukaryotes. Like the proteins of other RNA helicase families, they have a similar basic structure of the helicase core with motifs resembling those of other families, but are nevertheless clearly distinguishable from other families based on the conserved sequence motifs [5]. The Ski2-like helicase family has several representatives in one species, including, for example in yeast, the splicing factor Brr2, the Ski2 and Mtr4 proteins associated with exosome activities, and the ribosome-associated Ski2-like helicase 1, Slh1. These proteins are RNA-dependent ATPases that unwind duplex RNA in 3′–5′ direction with loading on a 3′ single-stranded extension [46]. The unwinding activity of Mtr4, a nuclear protein involved in RNA processing and degradation, is further stimulated if this helicase is present in the TRAMP (Trf4/Air2/Mtr4 polyadenylation) complex [47]. The Mtr4 helicase contains a ratchet domain that is highly conserved among Ski2-like RNA helicases and mutations within this domain abolish unwinding [48]. Recent single-molecule analysis of Mtr4 has shown that it is a 3′–5′ translocase that loads on a single-strand extension, senses the double-stranded RNA, and can perform several rounds of translocation and unwinding [49]. Interestingly, the unwound RNA does not snap-back to a dsRNA, and it has been suggested that the enzymes remains on the substrate and stops translocating once it can no longer sense a duplex ahead.

In addition to be involved in pre-mRNA splicing, proteins of the Ski2 family were found to be associated with RNA degradation. The Ski2 encoding gene from the yeast *Saccharomyces cerevisiae* was first described to have a superkiller phenotype if mutated, because in the absence of the Ski proteins, the double-stranded RNAs of killer strains is more abundant resulting in increased toxin production [50,51]. Later, we identified Mtr4, the nuclear homologue of Ski2, to be involved in pre-rRNA processing [52], and the Tartakoff laboratory identified the same gene in a screen for mutants accumulating poly(A)^+^ RNA in the nucleus [53]. A role in RNA degradation of Mtr4 was shown by the Tollervey laboratory who reported an association of Mtr4 with the polyadenylation complex (TRAMP), which stimulates RNA degradation by the exosome *in vitro* and *in vivo* [54]. Similarly, the cytoplasmic Ski2 is part of the Ski complex, composed of Ski2, Ski3, and Ski8 [55]. This complex involved in the mRNA surveillance process to assess the quality of transcripts is associated with the cytoplasmic exosome for RNA degradation [56,57]. Interestingly, as its bacterial counterpart, the cytoplasmic exosome can associate with the ribosome [58].

**Figure 2.**
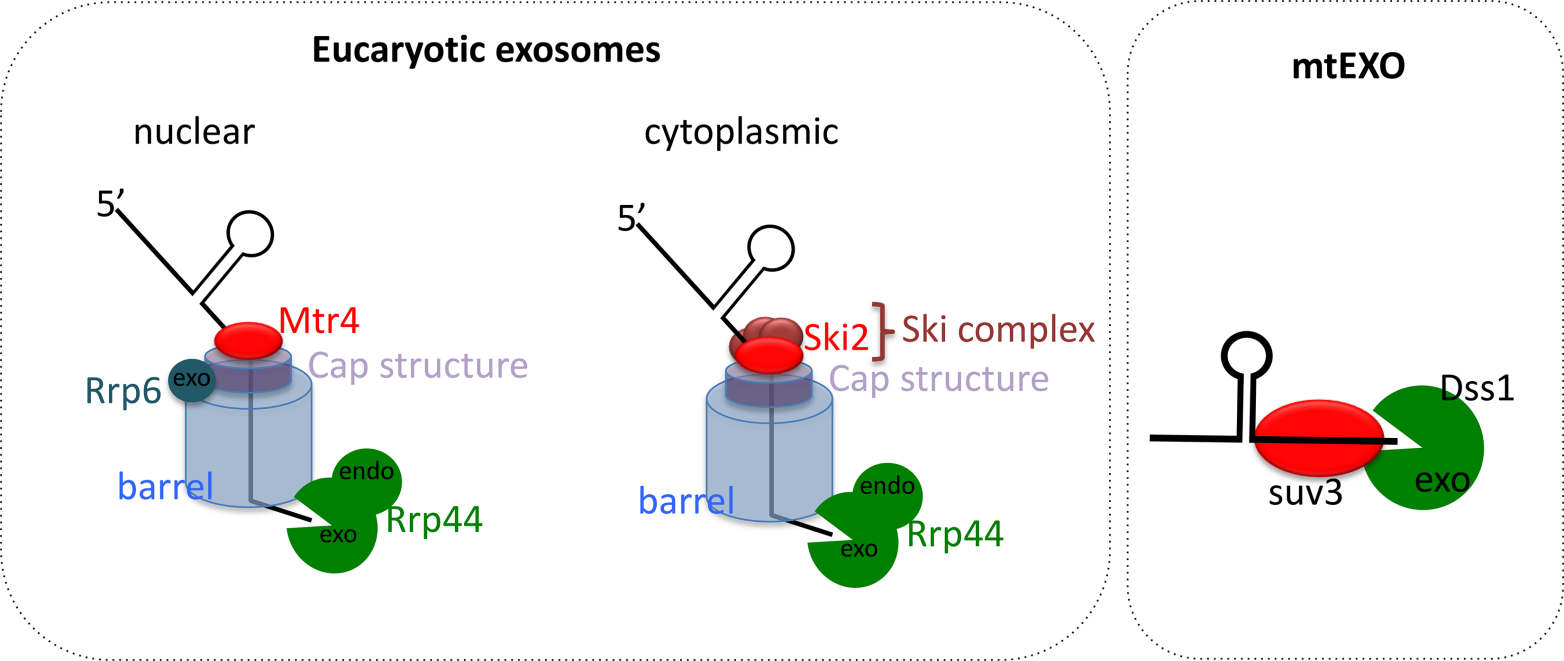
The exosome requires Ski2-like RNA helicases. The exosome barrel is composed of six subunit homologous to phosphorolytic nucleases, but without enzymatic activity in eukaryotes. This barrel forms a channel that can only accomodate ssRNA. The cap is composed of three proteins that contain RNA-binding domains. Rrp44 that bears the nucleolytic activity is at the exit of the channel. In the nucleus, the Mtr4 RNA helicase can alone unwind the RNA substrate or be associated with the polyadenylation complex TRAMP, NEXT, or PAXT. The nuclear exosome is also associated with a second exoribonuclease, Rrp6. In the cytoplasm, the exosome, which contains only Rrp44, is assisted by the Ski complex that contains the RNA helicase Ski2. Variations in the exosome and its cofactors have arisen in the course of evolution. As an example, archeal barrel of the exosome is composed of two subunits forming three identical heterodimers that show an overall similar organisation than the one observed in eukaryotes. But in contrast with the eukaryotic exosome barrel, the subunits bear catalytic activity in Archaea. In mitochondria, the Dss1–Suv3 proteins form the mtEXO degradosome that highlights the importance of the coordination between ribonucleases and helicases, as the activities of both proteins depend strongly on their interactions.

Eukaryotic cells have both a nuclear and a cytoplasmic exosome, which have the same basic structure, but are associated with different cofactors. The exosome is a large 9-subunit complex of a barrel-like structure composed of six subunits that show structural similarity to the bacterial PNPase and a cap composed of three proteins (Figure 2) [59]. This Exo9 complex is associated with the exoribonuclease Rrp44 (Exo10^44^) and in addition in the nuclear version with the exoribonuclease Rrp6 (Exo11^44/6^), which have hydrolytic 3′–5′ exonuclease activity [60]. The RNA to be degraded is introduced in a single-stranded form into the exosome channel. Cap proteins and the helicase protein sit at the top of the exosome barrel. The nuclear exosome is involved in processing of pre-rRNA, snoRNA, and snRNAs [61] and in the degradation of cryptic unstable transcripts [62]. Recently, Nop53 and Utp18 were identified as adapter proteins that interact with the arch domain of Mtr4 and provide specificity for the RNAs to be degraded by the exosome [63]. RNAs are also targeted to the exosome by the NEXT (nuclear exosome targeting complex, [64]) and PAXT (poly(A) tail exosome targeting, [65]) complexes. Although both cytoplasmic and nuclear exosomes contain Ski2-like helicases, the helicases used to feed the RNA into the channel of the barrel structure are not the same in the two compartments, as it was first shown in yeast with Ski2 in the cytoplasm and Mtr4 in the nucleus [54,56].

It is interesting to note that certain Archaea also possess an exosome with a similar structure to the eukaryotic complex [66,67]. In this complex, three heterodimers of the barrel structure contribute to the 3′–5′ exonuclease activity. Since the structure presents like in eukaryotes, a narrow channel for introducing the target RNA, it is likely that RNA helicases are required. Some Archaea also possess proteins similar to RNase J, opening the possibility that they also possess degradosome-like complexes [68,69].

## The nuclear-encoded Suv3 RNA helicase is required for mitochondrial RNA turnover

RNA turnover and the degradation of RNA fragments or splicing products are also important in mitochondria and chloroplasts. Several RNA helicases from the DEAD-box protein family and Suv3, an RNA helicase forming its own family most closely related to the Ski2-like RNA helicases in eukaryotes and purple bacteria [70], are required for gene expression in mitochondria. It was shown that yeast Suv3 associates with the Dss1 exoribonuclease to form a mitochondrial degradosome [71]. By *in vitro* reconstitution assays, it has been shown that Suv3 has a 3′–5′ helicase activity requiring a short 3′ single-stranded tail and that this helicase activity was dependent on the presence of Dss1, whereas the exonuclease showed basal activity alone that could be greatly stimulated by Suv3.

So far, little is known about RNA degradation and turnover in chloroplasts. These organelles contain RNase E and RNase J homologues, but so far no degradosome as such has been described. RNase E purified from *Arabidopsis* chloroplasts revealed an RNA-binding protein with similarity to transcription termination factor Rho, which is an RNA translocase [72] and may serve the purpose of RNA unwinding.

## Conclusion and perspectives

RNA turnover is crucial to maintain faithful gene expression and to allow cells to adapt to changing growth conditions, and in many instances, the inactivation of RNases causes strong growth defects. The structures of bacterial PNPases or eukaryotic exosomes require the insertion of single-stranded RNA into the complex, explaining the presence of RNA helicases to unwind inhibitory structures or to remove bound proteins. A crucial point in RNA turnover is the specificity that insures that a given RNA is degraded if necessary, but left alone if still useful to the cell. Although several adaptor proteins have been described for the different systems, the specificity of RNA turnover remains an important open question. Moreover, bacterial degradosomes are often associated with metabolic enzymes, but how they regulate degradosome activity is not known. In general, RNA helicases are not (very) substrate-specific; nevertheless, exchange of RNA helicases in bacterial degradosomes has been observed under different growth conditions. Future work will show why and how these RNA helicases are exchanged and function together with the RNA decay machineries.

An intriguing difference in the degradation of RNAs in bacterial and eukaryotic machineries is the differences in the RNA helicases. DEAD-box proteins, used in the bacterial degradosome, are non-processive RNA helicases that unwind locally secondary structures. In case of RhlB, the helicase is bound and stimulated by RNase E, which holds it on the substrate and will provide a certain artificial processivity. The role of Mtr4, and by analogy Ski2, may be more active in the process, since it has been shown that the RNA helicase translocates on the substrate to unwind the RNA. Thus, it remains to be seen to what extent the RNA helicases contribute to the active RNA degradation and the specificity of the decay complexes.

## Abbreviations

MTS: membrane-targeting sequence
PAXT: poly(A) tail exosome targeting
RBS: ribosome-binding sites
TRAMP: Trf4/Air2/Mtr4 polyadenylation
